# Effect of continuous or intermittent feeding of ergot contaminated grain in a mash or pelleted form on the performance and health of feedlot beef steers

**DOI:** 10.1093/jas/skae060

**Published:** 2024-03-05

**Authors:** Matthew R Reynolds, Kim Stanford, Daniela M Meléndez, Karen S Schwartzkopf-Genswein, Tim A McAllister, Barry R Blakley, John J McKinnon, Gabriel O Ribeiro

**Affiliations:** Department of Animal and Poultry Science, University of Saskatchewan, Saskatoon, SK, Canada; Biological Sciences Department, University of Lethbridge, Lethbridge, AB, Canada; Lethbridge Research and Development Centre, Agriculture and Agri-Food Canada, AB, Canada; Lethbridge Research and Development Centre, Agriculture and Agri-Food Canada, AB, Canada; Lethbridge Research and Development Centre, Agriculture and Agri-Food Canada, AB, Canada; Department of Veterinary Biomedical Sciences, University of Saskatchewan, Saskatoon, SK, Canada; Department of Animal and Poultry Science, University of Saskatchewan, Saskatoon, SK, Canada; Department of Animal and Poultry Science, University of Saskatchewan, Saskatoon, SK, Canada

**Keywords:** alkaloids, cattle, ergot, feedlot, intermittent feeding, pellet

## Abstract

This study evaluated the effect of feeding ergot contaminated grain continuously or intermittently through backgrounding (BG) and finishing (FN) in a mash or pelleted supplement on the growth performance, health and welfare parameters, and carcass characteristics of feedlot beef steers. Sixty black Angus steers (300 ± 29.4 kg BW) were used in a complete randomized 238-d study. Steers were stratified by weight and randomly assigned to four different diets (15 steers/treatment) and individually housed. Treatments included: (1) control [CON; no added ergot alkaloids (EA)], (2) continuous ergot mash (CEM; fed continuously at 2 mg total EA/kg of DM), (3) intermittent ergot mash (IEM; fed at 2 mg total EA/kg of DM, during the first week of each 21-d period and CON for the remaining 2 wk, this feeding pattern was repeated in each period), and (4) intermittent ergot pellet (IEP; fed at 2 mg of total EA/kg of DM as a pellet during the first week of each 21-d period and CON for the remaining 2 wk as described for IEM). Steers were fed barley based BG diets containing 40% concentrate:60% silage (DM basis) for 84 d (four 21-d periods), transitioned over 28 d (no ergot fed) to an FN diet (90% concentrate:10% silage DM basis) and fed for 126 d (six 21-d periods) before slaughter. In the BG phase, steer DMI (*P* < 0.01, 7.45 vs. 8.05 kg/d) and ADG (*P* < 0.01) were reduced for all EA diets compared to CON. The CEM fed steers had lower ADG (*P* < 0.01, 0.735 vs. 0.980 kg) and shrunk final BW (*P* < 0.01, 350 vs. 366 kg) than CON. CEM had lower gain:feed (*P* < 0.07, 0.130 vs. 0.142) than CON. In the FN phase, steer DMI (*P* < 0.01, 9.95 vs. 11.05 kg/d) and ADG (*P* = 0.04) were also decreased for all EA fed steers compared to CON. Total shrunk BW gain (*P *= 0.03, 202.5 vs. 225.2 kg), final BW (*P* = 0.03, 617.9 vs. 662.2 kg), and carcass weight (*P* = 0.06) decreased for all EA fed steers compared to CON. The percentage of AAA carcasses decreased for all EA fed steers (*P* < 0.01, 46.7 vs. 93.3%) compared to CON. EA fed steers had increased rectal temperatures (*P* < 0.01, 39.8 vs. 39.4 °C) compared to CON. Pelleting ergot contaminated grain did not reduce the impact of ergot alkaloids on any of the measured parameters during BG or FN. Continuously or intermittently feeding ergot contaminated diets (2 mg total EA/kg of DM) significantly reduced intake, growth performance, and carcass weight, with minimal impact on blood parameters in feedlot steers. Pelleting was not an effective method of reducing ergot toxicity.

## Teaser

This study evaluated the effect of continuous or intermittent feeding EA in a mash or pelleted form on growth performance, health and welfare parameters, and carcass characteristics of feedlot beef steers. Continuously or intermittently feeding an ergot contaminated diet (2 ppm EA) both reduced intake, growth performance, and carcass weight, but pelleting did not reduce the toxicities of EA.

## Introduction

Ergot is a disease that affects cereal grains found across the globe. Cereal ergot is produced by the fungus *Claviceps purpurea*, and the resulting ergot bodies contain toxic secondary metabolites named ergot alkaloids (EAs). Cereal ergot infections vary from year to year (Menzies and Turkington, 2015), but have shown an overall increase in prevalence since the late 1990s in western Canada (Walkowiak et al., 2022). This increased prevalence is a cause of concern for cattle producers as the consumption of EA can negatively affect the growth, welfare, and health of animals. The Canadian Food Inspection Agency (CFIA) recommends limiting EA in cattle diets to a maximum of 3 mg/kg. However, Blaney et al. (2009) and Sarich et al. (2023) have shown reduced beef cattle growth performance at EA concentrations less than 2 mg/kg. Signs of ergotism in feedlot cattle include reduction in feed intake and growth performance, vasoconstriction of the blood vessels, hyperthermia, as well as necrosis of extremities (ears, tails, and hooves) and death in extreme cases (Scott, 2009; Coufal-Majewski et al., 2016). Less common neurological symptoms such as convulsions can also occur (Craig et al., 2015). Vasoconstriction of blood vessels can lead to a reduction in the peripheral blood circulation, which can impair thermoregulation and reduce the oxygenation of tissues leading to gangrene with consequent loss of the ear tips, tail, and hooves (Blaney et al., 2009). Due to the toxicity of EA to cattle, developing strategies to reduce their effects would be valuable to livestock producers when feed options are restricted during periods of drought or when ergot-contaminated grain is abundant.

Pelleting EA contaminated grain was shown to reduce the concentrations of the toxins ergotamine and ergosine as compared to an ergot mash as a result of heating, increasing the conversion of the R-isomer to the less toxic S-isomer (Coufal-Majewski et al., 2017a). Grain deliveries occur weekly in feedlots and EA may not always be present continuously in the diet, but the extent to which cattle recover after a short period of EA exposure or conversely adapt to EA after continuous exposure is unknown. This has been shown in Pekin ducks where the most severe effects from EA on intake occurred in the beginning of feeding period but waned over time. However, poultry and swine seem to be less sensitive and may potentially have greater ability to adapt to EA compared to cattle (Mainka et al., 2005; Danicke, 2015). The objective of this study was to evaluate the effect of continuously or intermittently feeding a mash or pelleted cereal ergot-contaminated supplement on the growth performance, health, welfare parameters, and carcass characteristics of beef feedlot steers. It was hypothesized that continuously feeding EA to feedlot cattle would result in greater decrease of growth performance, health and welfare parameters, and carcass characteristics compared to intermittent feeding, and that cattle fed the pelleted EA supplement would experience reduced effects compared to the mash form.

## Materials and Methods

The use of steers in this study was reviewed and approved by Agriculture and Agri-Food Canada Lethbridge Research and Development Centre animal care committee (protocol number: 2105) with care and management following the guidelines of the Canadian Council on Animal Care (CCAC, 2009).

### Animals, diet, and experimental design

Sixty recently weaned black Angus steers with an initial body weight (BW) of 300 kg ± 29.4 kg were used in an 84-d backgrounding (BG) and 126-d finishing (FN) experiment, conducted between October 2021 and June 2022. Upon arrival, at the Agriculture and Agri-Food (AAFC) Lethbridge Research and Development Centre, feedlot steers were processed, vaccinated (Triangle 4 + PH-K, Boehringer Ingelheim Animal Health, Ingelheim am Rhein, Germany; Ultrabac 7/Somubac, Zoetis Canada Inc., Kirkland, QC, Canada), and treated for bovine respiratory disease (Draxxin, Zoetis Canada Inc., Kirkland, QC, Canada). At the start of the study (day 0), steers received a hormonal implant applied under the skin of the ear (Component TE-100; 100 mg trenbolone acetate, 10 mg estradiol, and 29 mg tylosin tartrate; Elanco Animal Health, Guelph, ON, Canada) to improve growth and gain:feed. Steers were re-implanted on day 113 with Component TE-200 (200 mg trenbolone acetate, 20 mg estradiol, and 29 mg tylosin tartrate; Elanco Animal Health, Guelph, ON, Canada).

Steers were blocked by BW and randomly assigned to one of four diets (15 steers/treatment) and randomly allocated to 60 individual pens (2.5 × 3 m) at the LRDC individual feeding barn. Each pen had a separate feeder with steers in adjacent pens sharing a heated water bowl. Steers had free access to water and were fed once daily at 0900 hours. Steers were fed a total mixed ration (TMR) using a Calan Data Ranger (American Calan Inc., Northwood, NH, USA). The control diet with no added ergot was mixed first and fed before the ergot diets, and the Data Ranger was cleaned daily after feeding by adding barley silage to it and letting it mix for 3 min before emptying. Feed ingredients, diets, and orts were collected once weekly. Diets included (1) control (CON; no added EA), (2) continuous ergot mash (CEM; fed continuously at 2 mg of total EA/kg of DM), (3) intermittent ergot mash (IEM; fed at 2 mg of total EA/kg of DM during the first week of each 21-d period and control diet for the remaining 2 wk), and (4) intermittent ergot pellet (IEP; fed at 2 mg of total EA/kg of DM incorporated into a pelleted supplement during the first week of each 21-d period and control diet for the other 2 wk).

Ergot-contaminated rye screenings were used as the source of ergot alkaloids. The ergot was added to the diet by replacing barley-grain in the supplement with ergot contaminated rye screenings. Dietary concentrations of ergot alkaloids were chosen based on responses observed in a previous feedlot study (Sarich et al., 2023). All diets were formulated to meet or exceed nutrient requirements of beef cattle (NASEM, 2016). Steers were fed 40% concentrate:60% barley-silage during BG and 90% concentrate:10% barley-silage during FN (DM basis). Monensin (Rumensin, Elanco Animal Health, Greenfield, IN) was included in all diets at a concentration of 33 mg/kg of DM to improve gain:feed. Rye ergot screenings, mash, and pellets (500 g) were sent to Romer Labs (Tulln, Austria) for analysis of R and S epimers of the six main alkaloids commonly associated with cereal ergot (i.e., ergocryptine, ergocornine, ergocristine, ergometrine, ergosine, and ergotamine) using HPLC-MS/MS (Malachová et al., 2014).

### Growth performance

Steers were weighed before feeding on two consecutive days at the start and end of the BG and FN phases, and after every 21-d. Individual BW were recorded, transformed to shrunk BW (BW × 0.96; NASEM, 2016) and used to determine ADG by regression. The gain efficiency (G:F) was calculated as ADG:DMI.

Feed amounts were recorded each morning and averaged by week. Weekly feed refusals were collected and weighed to estimate weekly DMI. Dry matter intake was calculated based on the number of days in each feeding period. Growth performance parameters such as ADG and gain:feed were estimated for the 84-d BG and 126-d FN phases. Rectal temperature was measured using a digital thermometer (GLA Agricultural Electronics, M750 Livestock Thermometer, San Luis Obispo, CA) on days 1, 42, 84, 112, 154, 196, and 236 as steers were being weighed.

### Infrared thermography and gait scores

Infrared thermography of the legs, ears, and tail were taken on day 1 and every 42 d until the end of the trial. Thermographic images were taken using an FLIR 140 infrared camera and processed with FLiR ThermCam QuickView 1.9.3 software (FLIR systems Inc., Burlington, ON, Canada) to identify changes in blood flow over the course of the study. Temperatures were analyzed as an average of three points across each extremity: at the tip, middle, and inside of both the left and right ears; the base, middle, and tip of the tail and at three points horizontally across the coronary band of the left front hooves. Only healthy hooves were assessed, as no lameness or hoof health issues were observed throughout the study. Ambient temperatures were recorded using six total iButton DS1923 temperature and humidity data loggers (Measurement Systems Ltd., Newbury, Berkshire, UK).

Gait scores were used to assess lameness and to identify progression of lameness. After leaving the squeeze chute during weigh days, steers were monitored for lameness as defined by gait scores greater than 2 according to the system described by Desrochers et al. (2001).

### Blood and hair analysis

Blood samples were collected from steers via jugular venipuncture using an 18 gauge 1″ needle into 10 mL non-additive vacutainer tubes (BD Vacutainer; Beckton Dickinson, Franklin Lakes, NJ) on the first day, middle, and at the end of the BG and FN phases after 0, 42, 84, 112, 154, 196, and 236 d. The blood samples were centrifuged (3,000 × rpm for 10 min at 4 °C) and the serum was collected and stored in 2 mL microcentrifuge vials at −40 °C until further analysis. Serum haptoglobin was analyzed using a colorimetric assay (Tridelta Development Ltd., Maynooth, Co., Kildare, Ireland). Prolactin was analyzed using an ELISA kit specifically for bovine prolactin (CEA846Bo, Cloud-Clone Corp., Katy, TX, USA). Serum chemistry was analyzed using an IDEXX VetTest Chemistry Analyzer model 8008 with software version 8.66A (IDEXX Laboratories, Inc., Westbrook, ME, USA) for albumin (ALB), alkaline phosphatase (ALKP), amylase (AMYL), blood urea nitrogen (BUN), alanine amino transferase (ALT), calcium (Ca), creatinine (CREA), gamma-glutamyl transpeptidase (GGT), globulin (GLOB), glucose (GLU), lipase (LIPA), total protein (TP), and total bilirubin (TBIL). Calibrations of the IDEXX VetTest Chemistry Analyzer were conducted monthly before each use with standards provided by the manufacturer to ensure proper precision and accuracy.

Blood samples were also collected and analyzed to determine complete blood cell count (CBC). Samples were collected into 6 mL EDTA tubes (BD vacutainer; Beckton Dickinson) for measurements of white blood cells (WBC), red blood cells (RBC), platelet count, and neutrophil:lymphocyte (N:L) ratio as an indicator of immune function using a HemaTrueHematology Analyzer (Heska, Loveland, Co).

Hair from the center of the forehead was clipped in a 10 × 10 cm square on days 0, 42, 84, 112, 154, 196, and 236 to determine hair cortisol concentrations as an indicator of chronic stress. Samples were stored at room temperature in sealed and labelled plastic bags then analyzed as described by Moya et al. (2013). Using a modified method of Moya et al. (2013), hair samples were dried under a fume hood, then 250 mg was weighed and washed and soaked for 3 min in 5 mL of isopropanol on a swirl shaker. Once fully dried, the samples were placed in a 10-mL metallic cylinder and ball ground using a 12-mm mill ball (Retsch Oscillating Mill MM 400 Mixer Mill, Newton, PA, USA) for 5 min at 22 rps (rotations per second). Then, 50 mg of ground sample was weighed into a 5-mL glass scintillation vial with 1.5 mL of methanol (EMD Chemicals Inc., Billerica, MA, USA) and incubated for 18 h at 100 rpm and 30 °C. The samples were evaporated using nitrogen in a RapidVap Vertex Evaporator (Labconco Corporation, Kansas City, MO, USA). Once evaporated they were analyzed using an Expanded Range High Sensitivity Salivary Cortisol Enzyme immunoassay kit (catalogue no. 1-30002, Salimetrics LLC, Penn State University, PA, USA).

### Carcass measurements

Carcass information was obtained from all steers at slaughter (Cargill Meat Solutions, High River, AB, Canada) and included hot carcass weight (HCW; with kidney, pelvic, and heart fat removed), dressing percentage, backfat thickness, ribeye area, saleable meat yield, and yield quality grades which were determined by qualified graders according to the Canadian Beef Grading Agency (Calgary, AB, Canada). Liver scores were graded as clear, minor, or severe according to the Elanco Liver Check System (Elanco Animal Health, Greenfield, IN).

### Feed sampling and chemical analysis

Feed ingredients, diets, and orts were sampled weekly, and oven dried at 55 °C for 7 d. Diet samples were composited by weigh period (21-d periods) and ground through a 1-mm screen (Wiley mill model 4, Thomas Scientific, Swedesboro, NJ). The ground diet samples were sent to Cumberland Valley Analytical Services (Waynesboro, PA, USA) for DM, crude protein (CP), neutral detergent fibre (NDF), acid detergent fibre (ADF), fat, starch, calcium, and phosphate analysis. The analytical DM concentration was determined according to AOAC (2000; method 930.15) in a drying oven at 135 °C. The CP concentration was determined (AOAC 2000; method 990.03) using a Leco FP-528 Nitrogen Combustion Analyzer (Leco, St. Joseph, MI). The ADF was determined according to AOAC (2000; method 973.18), and NDF was determined with amylase and sodium sulfite included using the method of Van Soest et al. (1991), with both analyses modified using a Whatman 934-AH glass micro-fiber filters with 1.5 µm particle retention in place of a fritted glass crucible. Fat was determined according to method 2003.05 (AOAC, 2006;) using a Tecator Soxtec System HT 1043 Extraction unit (Tecator, Foss, Eden Prairie, MN). The starch concentration was determined with correction for free glucose as described by Hall (2009). Calcium and phosphorus concentrations were determined according to AOAC (2000; method 985.01).

### Statistical analysis

Data were analyzed as a completely randomized design using the MIXED procedure of SAS (SAS Inst. Inc., Cary, NC) with steer as the experimental unit for all measurements and diet treatments included as fixed effect. The UNIVARIATE procedure of SAS was used to check data for normality of distribution and homogeneity of variance. The BG and FN phases were analyzed independently. Period was included as repeated measures for the analyses of dry matter intake, growth performance, prolactin, haptoglobin, hair cortisol, complete blood count, and general health blood panel data according to the following model:


$$\begin{array}{l} Yijk=\mu +T_{i}+P_{j}+TP_{ij}+e_{ijk} \end{array}$$

where *Yijk* is the observation, *µ* is the overall mean effect, T_*i*_ is the effect of diet treatment, *P*_*j*_ is the effect of sampling period, TP_*ij*_ is the effect of diet treatment × sampling period interactions, and *e*_*ijk*_ is the residual error. Various covariance structures were tested, and compound symmetry was selected based on having the lowest Akaike’s information criteria value. Granulocytes, lymphocytes, red blood cells, white blood cells, albumin, alkaline phosphatase, blood urea nitrogen, amylase, creatinine, and gamma-glutamyl data were not normally distributed and were log transformed before analysis. These variables in addition to the carcass quality grades and liver scores were analyzed using the GLIMMIX procedure of SAS. Infrared thermography, carcass quality grades, and liver scores data were analyzed according to the following model:


$$\begin{array}{l} Yij=\mu +T_{i}++e_{ij} \end{array}$$

where *Yij* is the observation, µ is the overall mean effect, *T*_*i*_ is the effect of diet treatment, and *e*_*ij*_ is the residual error.

Log transformed data were back transformed for reporting in tables. The treatment means were compared using the LSMEANS linear hypothesis test of SAS. The CONTRAST statement was utilized to compare the following dietary treatments: control vs. ergot (CON vs. CEM, IEM, and IEP), ergot continuous vs. ergot intermittent (CEM vs. IEM and IEP), and ergot mash vs. ergot pellet (IEM vs. IEP). Significance for all analyses is declared at *P* ≤ 0.05 and tendencies were considered when 0.05 < *P* ≤ 0.10.

## Results

### Ingredients, chemical composition, ergot alkaloid content

Minimal amounts of alkaloids were detected in the CON diets (0.05 mg/kg EA DM). The total EA (240.31 mg/kg) and the alkaloid profile present in the rye ergot screenings used to make the ergot diets is shown in **Table 1**. The total EA targeted in all EA diet treatments was 2 mg/kg. Pelleting the ergot supplement numerically increased the total EA concentration compared to the mash form, but estimated concentrations were highly variable (**Table 1**). Total EA measured in supplements were 0.00, 21.71, and 36.57 mg/kg for control mash, ergot mash, and ergot pellet, respectively. Analysis of EA in complete diets returned unrealistic low levels that did not align with the total EA in the ergot screenings used to make diet supplements and inclusion of the supplements in the diets (data not reported). Ergocristine/inine and ergosine/inine were the alkaloids measured with the greatest and least concentration in the ergot screenings and diet supplements, respectively. The alkaloid with the second greatest content was ergotamine/inine. Increasing levels of EA did not appear to affect the overall nutrient composition of the diets (**Table 2**).

**Table 1.** Diet supplement composition, and ergot alkaloid profile (mean ± SD) of ergot screenings and supplements

Item	Ergot screenings	Control mash	Ergot mash	Ergot pellet
Ingredients (% of DM)				
Barley chop	—	55.73	38.93	38.93
Calcium carbonate	—	24.66	24.66	24.66
Rye ergot screenings	100	0.00	16.80	16.80
Canola meal	—	9.86	9.86	9.86
Iodized salt	—	2.96	2.96	2.96
Molasses	—	2.47	2.47	2.47
Urea	—	1.97	1.97	1.97
Vitamin premix	—	0.99	0.99	0.99
Canola oil	—	0.99	0.99	0.99
Vitamin E	—	0.07	0.07	0.07
Rumensin 200	—	0.31	0.31	0.31
Ergot Alkaloids (mg/kg)				
Ergocornine	11.80 ± 4.70	0.00 ± 0.00	0.63 ± 0.00	1.44 ± 0.58
Ergocorninine	3.82 ± 1.50	0.00 ± 0.00	0.28 ± 0.11	0.49 ± 0.20
Ergocristine	103.00 ± 41.00	0.01 ± 0.00	11.20 ± 4.50	18.00 ± 7.20
Ergocristinine	19.60 ± 7.80	0.00 ± 0.00	2.49 ± 1.00	4.10 ± 1.60
alpha-Ergocryptine	17.80 ± 7.10	0.00 ± 0.00	1.56 ± 0.63	2.78 ± 1.10
alpha-Ergocryptinine	4.30 ± 1.30	0.00 ± 0.00	0.49 ± 0.15	0.74 ± 0.22
Ergometrine	15.40 ± 6.20	0.00 ± 0.00	0.42 ± 0.17	0.37 ± 0.15
Ergometrinine	3.20 ± 1.30	0.00 ± 0.00	0.06 ± 0.03	0.09 ± 0.04
Ergosine	6.67 ± 2.70	0.00 ± 0.00	0.66 ± 0.26	0.91 ± 0.36
Ergosinine	1.72 ± 0.52	0.00 ± 0.00	0.16 ± 0.05	0.27 ± 0.08
Ergotamine	45.00 ± 18.00	0.00 ± 0.00	2.95 ± 1.20	6.08 ± 2.40
Ergotaminine	8.00 ± 2.40	0.00 ± 0.00	0.80 ± 0.24	1.23 ± 0.37
Total alkaloids (mg/kg)	240.31 ± 94.52	0.01 ± 0.00	21.71 ± 8.58	36.50 ± 14.30
Total R-epimers (mg/kg)	199.67 ± 79.70	0.01 ± 0.00	17.43 ± 7.01	29.58 ± 11.79
Total S-epimers (mg/kg)	40.64 ± 14.82	0.00 ± 0.00	4.28 ± 1.57	6.92 ± 2.51

**Table 2.** Ingredients and chemical composition (Mean ± SD) of backgrounding (BG) and finishing (FN) experimental diets

	Backgrounding				Finishing			
Item	CON^1^	CEM^2^	IEM^3^	IEP^4^	CON	CEM	IEM	IEP
Diet ingredient^5^, % of DM								
Barley grain	30.00	30.00	30.00	30.00	82.50	82.50	82.50	82.50
Barley silage	60.00	60.00	60.00	60.00	10.00	10.00	10.00	10.00
Canola meal	5.00	5.00	5.00	5.00	2.50	2.50	2.50	2.50
Supplement^6^	5.00	5.00	5.00	5.00	5.00	5.00	5.00	5.00
Chemical composition, % of DM								
Dry matter	49.9 ± 2.47	49.3 ± 2.73	50.3 ± 1.98	49.4 ± 2.41	77.4 ± 1.73	77.8 ± 1.01	77.6 ± 1.56	77.6 ± 1.06
Crude protein	13.2 ± 0.14	13.6 ± 0.22	13.6 ± 0.11	13.4 ± 0.26	14.0 ± 0.23	13.8 ± 0.32	13.9 ± 0.33	14.1 ± 0.38
Acid detergent fiber	25.0 ± 0.28	25.9 ± 0.06	24.7 ± 0.61	24.4 ± 0.51	14.0 ± 0.61	13.8 ± 0.51	13.1 ± 0.79	13.5 ± 0.54
Neutral detergent fiber	39.3 ± 0.28	39.7 ± 0.25	38.4 ± 0.57	38.1 ± 0.84	27.7 ± 1.41	26.7 ± 0.88	26.7 ± 1.07	25.6 ± 0.65
Starch	26.1 ± 0.32	24.5 ± 0.29	26.4 ± 0.66	27.0 ± 1.12	39.6 ± 1.56	40.3 ± 0.43	42.0 ± 1.65	40.0 ± 1.09
Total digestible nutrients	69.3 ± 0.63	69.2 ± 0.51	69.8 ± 0.42	68.7 ± 0.77	73.7 ± 0.75	74.2 ± 0.32	75.1 ± 0.01	74.8 ± 0.44
NE_m_, Mcal/kg of DM^7^	1.71 ± 0.02	1.71 ± 0.02	1.74 ± 0.02	1.70 ± 0.02	1.89 ± 0.03	1.90 ± 0.03	1.94 ± 0.03	1.93 ± 0.03
NE_g_, Mcal/kg of DM^7^	1.10 ± 0.02	1.10 ± 0.02	1.12 ± 0.02	1.09 ± 0.02	1.25 ± 0.03	1.27 ± 0.01	1.29 ± 0.01	1.29 ± 0.02
Calcium	0.66 ± 0.03	0.61 ± 0.04	0.72 ± 0.03	0.73 ± 0.05	0.64 ± 0.06	0.58 ± 0.07	0.51 ± 0.03	0.72 ± 0.08
Phosphate	0.28 ± 0.02	0.28 ± 0.01	0.29 ± 0.00	0.29 ± 0.01	0.39 ± 0.02	0.39 ± 0.07	0.39 ± 0.02	0.39 ± 0.02

^1^CON = Control, no added ergot alkaloids (EA).

^2^CEM = Continuous ergot mash, fed continuously 2 mg total EA/kg of diet DM.

^3^IEM = Intermittent ergot mash, fed intermittently 2 mg total EA/kg of diet DM during the first week of each 21-d period and CON for the remaining 2 wk.

^4^IEP = Intermittent ergot pellet, fed intermittently 2 mg total EA/kg of diet DM incorporated into a pelleted supplement during the first week of each 21-d period and CON for the remaining 2 wk.

^5^DM, dry matter.

^6^Supplements composition described in Table 1. Ergot diets formulated to provide 2 mg of total EA/kg of diet DM.

^7^NEm and NEg were estimated according to NASEM (2016).

### Feed intake and growth performance

During the BG phase, all EA fed steers had decreased (*P* = 0.01) shrunk total BW gain, DMI, and ADG compared to CON (**Table 3**). No differences (*P* ≥ 0.20) among treatments were observed for shrunk initial weight or shrunk final weight. However, a tendency for a decrease in gain:feed was shown for EA fed steers compared to CON (*P *= 0.06). There was no effect on intake or growth performance of BG steers from feeding cereal EA continuously compared to intermittently or by feeding it in a pelleted vs. a mash form.

**Table 3.** Effect of continuous or intermittent feeding of ergot contaminated grain in a mash or pelleted form on intake and growth performance of backgrounding (BG) and finishing (FN) feedlot steers

	Treatments					*P*-value		
	CON^1^	CEM^2^	IEM^3^	IEP^4^	SEM	CON vs. Ergot	Cont. vs. Int.	Ergot Mash vs. Ergot Pellet
Backgrounding								
Shrunk initial BW, kg	289	288	290	289	7.37	0.97	0.91	0.91
Shrunk final BW, kg	366	350	354	351	9.40	0.20	0.81	0.58
Shrunk total BW gain, kg	77.3	61.7	64.6	62.3	4.73	0.01	0.77	0.21
DMI, kg/d	8.05	7.31	7.48	7.55	0.182	0.01	0.55	0.42
ADG, kg	0.98	0.74	0.77	0.74	0.056	0.01	0.77	0.21
Gain:Feed	0.142	0.130	0.134	0.130	0.0051	0.06	0.99	0.27
Finishing								
Shrunk initial BW, kg	446	414	426	419	8.51	0.01	0.43	0.64
Shrunk final BW, kg	671	618	648	628	11.51	<0.01	0.19	0.87
Shrunk total BW gain, kg	225.2	202.5	221.7	208.7	5.66	0.03	0.09	0.63
DMI, kg/d	11.05	9.95	10.26	9.96	0.253	<0.01	0.61	0.12
ADG, kg	1.80	1.62	1.77	1.67	0.045	0.04	0.08	0.60
Gain:feed	0.161	0.163	0.173	0.168	0.0034	0.09	0.08	0.02

^1^CON = Control, no added ergot alkaloids (EA).

^2^CEM = Continuous ergot mash, fed continuously 2 mg total EA/kg of diet DM.

^3^IEM = Intermittent ergot mash, fed intermittently 2 mg total EA/kg of diet DM during the first week of each 21-d period and CON for the remaining 2 wk.

^4^IEP = Intermittent ergot pellet, fed intermittently 2 mg total EA/kg of diet DM incorporated into a pelleted supplement during the first week of each 21-d period and CON for the remaining 2 wk.

Shrunk BW = BW × 0.96.

During the FN phase, shrunk initial BW (*P =* 0.01), final BW (*P <* 0.01), shrunk total BW gain (*P = *0.03), DMI (*P* < 0.01), and ADG (*P =* 0.04) were lower for all EA fed steers compared to CON. The gain:feed was greater for steers fed intermittent ergot mash compared to the pellet (*P *= 0.02). There was no observed effect of feeding EA continuously compared to intermittently on intake and growth performance of FN feedlot steers.

### Blood, hair, and infrared image analysis

During the BG, EA fed steers had greater rectal temperature (*P* = 0.01) and lower blood serum albumin (*P* = 0.02), alkaline phosphatase (*P* = 0.04), and globulin (*P* = 0.04) compared to CON fed steers (**Table 4**). Steers fed EA intermittently had lower blood serum albumin (*P* < 0.01), amylase (*P* = 0.01), calcium (*P* = 0.05), gamma-glutamyl transferase (*P* = 0.01), globulin (*P* = 0.01), and total protein (*P* < 0.01) compared to steers fed EA continuously. Feeding EA in a pellet increased (*P* = 0.02) blood serum GGT and tended to increase creatinine (*P* = 0.08) levels when compared to feeding EA in a mash. Furthermore, serum prolactin, serum haptoglobin, and hair cortisol were not affected (*P* ≥ 0.15) by EA in the diets. Red blood cell counts increased in steers fed EA continuously compared to intermittently (*P =* 0.04). However, no other treatment effects were observed (*P* ≥ 0.11) for other CBC parameters.

**Table 4.** Effect of continuous or intermittent feeding of ergot contaminated grain in a mash or pelleted form on rectal temperature, hair cortisol, and blood chemistry of backgrounded (BG) steers

	Treatments					*P*-value		
	CON^1^	CEM^2^	IEM^3^	IEP^4^	SEM	CON vs. Ergot	Cont. vs. Int.	Ergot mash vs. ergot pellet
Rectal temp, °C	39.43	39.57	39.56	39.52	0.020	0.01	0.83	0.23
Hair cortisol, pg/mg	2.49	2.36	2.55	2.18	0.085	0.54	0.99	0.15
Prolactin, ng/ml	27.10	23.46	30.03	27.18	0.661	0.25	0.61	0.61
Haptoglobin, mg/ml	0.07	0.09	0.08	0.07	0.632	0.34	0.43	0.63
Blood health panel								
Albumin^—^ALB, g/L	29.03	28.77	27.38	26.74	1.008	0.02	<0.01	0.33
Alkaline phosphatase—ALP, U/L	138.32	124.42	119.42	107.67	0.016	0.04	0.92	0.24
Amylase—AMYL, IU	75.57	78.91	65.46	66.18	1.924	0.24	0.01	0.90
Blood urea nitrogen—BUN, mmol/L	2.56	2.52	2.32	2.21	1.019	0.20	0.16	0.65
Alanine transaminase—ALT, U/L	59.58	62.83	59.03	59.24	0.964	0.73	0.13	0.94
Calcium—Ca, mmol/L	2.45	2.48	2.33	2.38	0.376	0.38	0.05	0.45
Creatinine—CREA, umol/L	91.13	97.60	96.91	88.11	0.912	0.45	0.24	0.08
Gamma-glutamyl transferase—GGT, IU	24.32	25.32	21.31	23.94	0.017	0.37	0.01	0.02
Globulin—GLOB, g/L	34.33	35.62	32.36	32.42	0.458	0.04	0.01	0.93
Glucose—GLU, mmol/L	5.28	2.31	5.04	5.17	0.065	0.50	0.22	0.51
Lipase—LIPA, IU	62.25	57.10	52.07	58.96	2.651	0.34	0.81	0.38
Total protein—TP, g/L	62.98	64.69	59.99	59.90	0.615	0.33	<0.01	0.96
Total bilirubin—TBIL, umol/L	2.30	2.37	2.31	2.21	0.990	0.99	0.70	0.75
Complete blood count								
Granulocytes, 10^3^ µL	2.42	2.34	2.35	2.46	0.009	0.78	0.61	0.47
Lymphocytes, 10^3^ µL	6.44	6.25	6.46	6.49	0.038	0.78	0.11	0.84
Monocytes, 10^3^ µL	0.81	0.80	0.61	0.80	0.011	0.89	0.87	0.86
Platelets, 10^3^ µL	314.39	331.65	313.79	327.90	3.270	0.30	0.29	0.25
Red blood cells, 10^3^ µL	8.96	9.06	8.82	8.81	0.028	0.59	0.04	0.66
White blood cells, 10^3^ µL	9.82	9.55	9.72	9.93	0.073	0.73	0.32	0.51

^1^CON = Control, no added ergot alkaloids (EA).

^2^CEM = Continuous ergot mash, fed continuously 2 mg total EA/kg of diet DM.

^3^IEM = Intermittent ergot mash, fed intermittently 2 mg total EA/kg of diet DM during the first week of each 21-d period and CON for the remaining 2 wk.

^4^IEP = Intermittent ergot pellet, fed intermittently 2 mg total EA/kg of diet DM incorporated into a pelleted supplement during the first week of each 21-d period and CON for the remaining 2 wk.

During the FN phase, greater rectal temperature (*P* < 0.01) and lower blood serum amylase (*P* < 0.01), urea nitrogen (*P =* 0.01), and lipase (*P *< 0.01) were observed for all EA fed steers compared to CON (**Table 5**). Additionally, an increase in serum amylase (*P* < 0.01) and decrease in GGT (*P = *0.04) was observed for steers fed mashed vs. pelleted EA. A tendency for decreased RBC was observed for steers fed EA continuously vs. intermittently (*P *= 0.09). However, the remainder of CBC parameters was not affected by EA. A decrease in hair cortisol was observed for FN steers feed EA continuously compared to intermittently (*P *< 0.01), and in mashed vs. pelleted supplement (*P *= 0.02). A tendency was observed for decreased serum haptoglobin for all EA fed steers compared to CON (*P *= 0.09). Similar to BG, serum prolactin was not affected by EA inclusion during FN.

**Table 5.** Effect of continuous or intermittent feeding of ergot contaminated grain in a mash or pelleted form on rectal temperature, hair cortisol, and blood chemistry of finishing (FN) steers

	Treatments					*P*-value		
	CON^1^	CEM^2^	IEM^3^	IEP^4^	SEM	CON vs. Ergot	Cont. vs. Int.	Ergot mash vs. ergot pellet
Rectal temp, °C	39.40	39.78	39.73	39.55	0.016	<0.01	0.09	0.06
Hair cortisol, pg/mg	1.76	1.78	1.55	1.23	1.035	0.05	<0.01	0.02
Prolactin, ng/ml	35.04	35.24	38.77	33.05	1.084	0.95	0.95	0.61
Haptoglobin, mg/ml	0.13	0.11	0.11	0.10	1.034	0.09	0.59	0.63
Blood health panel								
Albumin—ALB, g/L	25.31	24.29	23.43	25.31	0.021	0.42	0.96	0.20
Alkaline phosphatase—ALP, U/L	101.81	92.00	99.82	111.79	1.729	0.92	0.18	0.33
Amylase—AMYL, IU	61.87	53.89	51.14	46.68	0.061	<0.01	0.42	<0.01
Blood urea nitrogen—BUN, mmol/L	4.10	3.79	3.48	3.49	0.071	0.01	0.16	0.97
Alanine transaminase—ALT, U/L	61.42	60.54	58.04	56.99	0.947	0.13	0.27	0.74
Calcium—Ca, mmol/L	2.13	2.05	1.96	2.09	0.029	0.20	0.76	0.16
Creatinine—CREA, umol/L	84.47	88.74	80.84	82.23	0.630	0.92	0.20	0.83
Gamma-glutamyl transferase—GGT, IU	27.85	28.34	23.36	28.52	0.591	0.57	0.25	0.04
Globulin—GLOB, g/L	1.43	1.45	1.45	1.44	0.458	0.95	0.67	0.60
Glucose—GLU, mmol/L	4.78	4.31	4.52	4.69	0.092	0.30	0.27	0.57
Lipase—LIPA, IU	37.29	34.61	29.24	28.61	0.087	<0.01	0.06	<0.01
Total protein—TP, g/L	56.17	55.41	54.03	54.76	0.823	0.53	0.67	0.79
Total bilirubin—TBIL, µmol/L	3.18	2.71	2.67	2.70	0.938	0.94	0.06	0.12
Complete blood count								
Granulocytes, 10^3^ µL	3.36	3.11	3.20	3.14	0.108	0.28	0.75	0.79
Lymphocytes, 10^3^ µL	5.98	5.74	6.14	6.00	1.006	0.91	0.11	0.56
Monocytes, 10^3^ µL	0.93	0.89	0.93	0.90	0.008	0.60	0.33	0.52
Platelets, 10^3^ µL	262.93	268.68	249.95	263.82	1.834	0.85	0.33	0.33
Red blood cells, 10^6^ µL	8.97	9.05	8.85	8.72	1.002	0.48	0.09	0.46
White blood cells, 10^3^ µL	10.35	9.86	10.40	10.18	1.005	0.54	0.22	0.60

^1^CON = Control, no added ergot alkaloids (EA).

^2^CEM = Continuous ergot mash, fed continuously 2 mg total EA/kg of diet DM.

^3^IEM = Intermittent ergot mash, fed intermittently 2 mg total EA/kg of diet DM during the first week of each 21-d period and CON for the remaining 2 wk.

^4^IEP = Intermittent ergot pellet, fed intermittently 2 mg total EA/kg of diet DM incorporated into a pelleted supplement during the first week of each 21-d period and CON for the remaining 2 wk.

At the end of BG, the temperature in both ears was lower (*P *= 0.01) in steers fed EA as a pellet compared to a mash (**Table 6**). No other treatment effects were observed for the infrared image measurements made during BG.

**Table 6.** Effect of continuous or intermittent feeding of ergot contaminated grain in a mash or pelleted form on ear, hoof, and tail temperatures of backgrounded (BG) steers

	Treatments					*P*-value		
	CON^1^	CEM^2^	IEM^3^	IEP^4^	SEM	CON vs. Ergot	Cont. vs. Int.	Ergot mash vs. ergot pellet
BG start of Period 1—AVG (October 13/21—start of BG)								
Both ears, °C	17.01	16.22	16.21	16.19	0.497	0.52	0.99	0.99
Left ear, °C	18.00	18.83	17.92	17.39	0.026	0.97	0.43	0.76
Right ear, °C	17.43	17.56	17.18	16.99	0.029	0.88	0.72	0.90
Hoof, °C	19.14	19.63	18.83	19.51	0.032	0.87	0.69	0.61
Tail, °C	17.96	18.36	17.31	17.14	0.044	0.62	0.15	0.85
BG end of Period 2—AVG (November 24/21, 2 wk off Int.)								
Both Ears, °C	13.12	12.62	13.46	13.17	0.573	0.98	0.63	0.88
Left Ear, °C	14.92	13.45	14.53	14.38	0.027	0.59	0.55	0.94
Right Ear, °C	11.32	11.79	12.39	11.97	0.031	0.57	0.79	0.82
Hoof, °C	15.29	13.05	15.22	13.22	0.033	0.24	0.39	0.27
Tail, °C	12.00	11.01	10.26	9.87	0.040	0.11	0.40	0.79
BG end of Period 4—AVG (January 5/22, 2 wk off Int.)								
Both ears, °C	7.03	4.05	7.41	3.26	0.030	0.08	0.31	0.01
Left ear, °C	7.98	5.02	8.74	4.75	0.023	0.25	0.30	0.04
Right ear, °C	6.07	3.09	6.09	1.77	0.028	0.06	0.53	0.01
Hoof, °C	9.42	7.99	8.62	9.94	0.028	0.65	0.34	0.39
Tail, °C	5.87	4.58	3.60	4.95	0.031	0.19	0.81	0.34

^1^CON = Control, no added ergot alkaloids (EA).

^2^CEM = Continuous ergot mash, fed continuously 2 mg total EA/kg of diet DM.

^3^IEM = Intermittent ergot mash, fed intermittently 2 mg total EA/kg of diet DM during the first week of each 21-d period and CON for the remaining 2 wk.

^4^IEP = Intermittent ergot pellet, fed intermittently 2 mg total EA/kg of diet DM incorporated into a pelleted supplement during the first week of each 21-d period and CON for the remaining 2 wk.

A decrease in temperature of the tail was observed for all EA fed steers (*P* = 0.02) compared to CON at the beginning of FN (period 1; **Table 7**). A tendency (*P* = 0.09) for a decrease in tail temperature was observed for all EA fed steers compared to CON at the end of period 2 of FN. There was also tendency (*P* = 0.10) for hoof temperature to decline at the end of period 4 of FN and at the end of the study (period 7; *P* = 0.09) for all EA fed steers compared to CON. At the end of period 2 during FN, an increase in ear temperature was observed for continuously vs. intermittently EA fed steers (*P* = 0.03), with a similar tendency (*P* = 0.06) being observed in period 6. An increase (*P* = 0.04) in tail temperature was observed at the end of the study (FN period 7) for continuously vs. intermittently EA fed steers. A tendency for ear temperature to decrease (*P* = 0.09) was observed at the beginning of the FN phase (period 1) and at the end of period 2 (*P* = 0.04) for steers fed EA mash compared to the EA pellet.

**Table 7.** Effect of continuous or intermittent feeding of ergot contaminated grain in a mash or pelleted form on ear, hoof, and tail temperatures of finishing (FN) steers

	Treatments					P-value		
	CON^1^	CEM^2^	IEM^3^	IEP^4^	SEM	Ctrl vs. Ergot	Cont. vs. Int.	Ergot mash vs. ergot pellet
FN start of Period 1—AVG (February 2/22—start of FN)								
Both ears, °C	7.46	8.98	8.86	6.23	0.029	0.65	0.28	0.09
Left ear, °C	9.54	9.94	10.33	7.72	0.026	0.88	0.53	0.13
Right ear, °C	5.40	7.51	7.36	4.72	0.026	0.42	0.33	0.13
Hoof, °C	0.26	-0.08	0.11	-0.27	0.047	0.61	1.00	0.63
Tail, °C	6.66	4.45	5.29	3.56	0.391	0.02	0.98	0.13
FN end of Period 2—AVG (March 16/22, 2 wk off Int.)								
Both ears, °C	19.46	19.80	18.68	16.11	0.038	0.35	0.03	0.04
Left ear, °C	19.83	20.22	18.79	17.13	0.030	0.44	0.06	0.21
Right ear, °C	19.10	19.38	18.57	15.05	0.036	0.33	0.04	0.01
Hoof, °C	17.64	18.90	19.07	18.13	0.033	0.51	0.82	0.51
Tail, °C	17.60	15.56	15.49	15.84	0.045	0.09	0.91	0.74
FN end of Period 4—AVG (April 27/22, 2 wk off Int.)								
Both ears, °C	17.85	19.31	18.73	18.25	0.392	0.33	0.42	0.67
Left ear, °C	18.52	19.16	19.49	18.37	0.022	0.63	0.84	0.36
Right ear, °C	17.09	19.38	17.65	18.13	0.035	0.18	0.24	0.89
Hoof, °C	23.99	23.23	23.01	21.65	0.345	0.10	0.31	0.18
Tail, °C	20.53	20.77	18.83	20.37	0.041	0.51	0.19	0.13
FN end of Period 6—AVG (June 8/22, 2 wk off Int.)								
Both ears, °C	22.64	23.02	22.35	21.71	0.001	0.56	0.06	0.27
Left ear, °C	21.18	23.23	22.90	22.29	0.310	0.45	0.24	0.31
Right ear, °C	22.07	22.78	21.81	21.17	0.055	0.77	0.03	0.32
Hoof, °C	27.25	26.45	25.62	25.65	0.043	0.09	0.34	0.97
Tail, °C	23.59	24.14	23.23	23.03	0.059	0.76	0.04	0.71

^1^CON = Control, no added ergot alkaloids (EA).

^2^CEM = Continuous ergot mash, fed continuously 2 mg total EA/kg of diet DM.

^3^IEM = Intermittent ergot mash, fed intermittently 2 mg total EA/kg of diet DM during the first week of each 21-d period and CON for the remaining 2 wk.

^4^IEP = Intermittent ergot pellet, fed intermittently 2 mg total EA/kg of diet DM incorporated into a pelleted supplement during the first week of each 21-d period and CON for the remaining 2 wk.

### Carcass characteristics

Carcass weights tended to decrease (*P =* 0.06) for all EA fed steers compared to CON. No treatment effects were observed for carcass dressing percentage (*P* ≥ 0.73) (**Table 8**). Carcass backfat thickness had a tendency to decrease (*P =* 0.08) for steers fed EA compared to CON and also decreased for steers fed EA continuously versus intermittently (*P = *0.05) and for steers fed EA as a pellet compared to a mash (*P =* 0.01). A tendency for smaller ribeye area (*P* = 0.09) was observed for EA fed steers compared to CON. Carcass yield scores were lower (*P = *0.01) and lean meat yield (*P = *0.03) was greater for all EA fed steers compared to CON (**Table 8**). Consequently, a decrease in the percentage of carcasses classified as AAA, and a tendency for increase in AA was observed for all EA fed steers (*P < *0.01) compared to CON. Neither the number nor severity of liver abscesses were affected by EA.

**Table 8.** Effect of continuous or intermittent feeding of ergot contaminated grain in a mash or pelleted form on carcass quality and liver abscesses of finishing (FN) steers

	Treatments					*P*-value		
	CON^1^	CEM^2^	IEM^3^	IEP^4^	SEM	CON vs. Ergot	Cont. vs. Int.	Ergot mash vs. ergot pellet
Carcass weight, kg	401.12	375.79	394.90	380.16	7.21	0.06	0.23	0.91
Dressing percentage^5^, %	60.60	60.82	60.91	60.53	0.06	0.73	0.83	0.98
Backfat thickness, mm	18.35	17.85	15.03	14.51	0.03	0.08	0.05	0.01
Rib-eye area, cm	80.38	85.99	87.11	82.72	0.01	0.09	0.73	0.50
Lean meat yield, %	50.46	52.63	54.32	52.69	0.03	0.03	0.52	0.08
Marbling score	437.53	391.85	417.20	416.21	6.65	0.07	0.16	0.88
Yield score	4.25	3.70	3.53	3.66	0.06	0.01	0.68	0.07
Quality grade^6^, %								
AAA	93.33	46.67	66.67	60.00	—	<0.01	0.44	0.70
AA	6.67	46.67	33.33	40.00	—	0.07	0.39	0.22
Liver score^7^, %								
Abscessed livers, %	46.67	40.00	40.00	33.33	—	0.57	0.68	0.53
Minor abscesses, %	26.67	33.33	20.00	13.33	—	0.66	0.16	0.21
Severely abscessed, %	20.00	6.67	20.00	20.00	—	0.60	0.32	0.44

^1^CON = Control, no added ergot alkaloids (EA).

^2^CEM = Continuous ergot mash, fed continuously 2 mg total EA/kg of diet DM.

^3^IEM = Intermittent ergot mash, fed intermittently 2 mg total EA/kg of diet DM during the first week of each 21-d period and CON for the remaining 2 wk.

^4^IEP = Intermittent ergot pellet, fed intermittently 2 mg total EA/kg of diet DM incorporated into a pelleted supplement during the first week of each 21-d period and CON for the remaining 2 wk.

^5^Dressing percentage was determined by dividing the carcass weight by the body weight measured at the end of the study after correction (4%) for shrink.

^6^Quality grades were determined according to Canadian Beef Grading Agency and expressed as a percentage of total carcasses.

^7^Liver scores classified as clear, minor, or severe adapted by the Elanco Liver Check System (Elanco Animal Health, Greenfield).

## Discussion

There is limited research about the production and health impact of the consumption of cereal EA produced by the fungus *Claviceps purpurea* in feedlot cattle. Over 80 types of ergot alkaloids have been isolated (Schiff, 2006), but most research has focused on the alkaloids associated with endophyte-infected tall-fescue (Thompson and Stuedemann, 1993; Duckett et al., 2016; Alfaro et al., 2021). Given the differences between fescue and cereal ergot alkaloids, this study focused on ergocornine, ergocristine, ergocryptine, ergometrine, ergosine, and ergotamine, the main alkaloids in cereal ergot (Grusie et al., 2018).

### Ergot alkaloid content and pelleting

The EA diets were formulated to achieve 2 mg of EA/kg of diet DM. The diet-measured concentration of total dietary EA was lower than calculated based on the EA concentration of the rye screenings in both the BG and FN diets. This inconsistency is likely due to issues with EA analysis of complete diets, but the lack of representative diet sampling may have also contributed to this problem. Stanford et al. (2022) and Sarich et al. (2023) have reported similar inconsistencies in EA analysis. Increased particle size and inconsistent sample size has been reported to increase variation and error in analyses of EA concentration (Grusie et al., 2017). Pelleting may increase palatability and feed consumption while reducing the ability of cattle to sort their feed (Williams et al., 2008; Huang et al., 2015). However, pelleting did not reduce the overall concentrations of EA in the supplement in the present study, in contrast to what was reported by Coufal-Majewski et al. (2017b). Instead, pelleting appeared to increase concentrations of EA compared to mash supplement, similar to results reported by Stanford et al. (2022). This was believed to be an artifact of the pelleting process as the pressure and heat during pelleting may make the EA more available for extraction during the EA assay (Stanford et al., 2022). Although the pellets numerically had greater measured EA concentration, we believe the actual physical concentrations in the EA mash and pellet were similar as the observed effects in steers were similar.

### Intake and growth performance

Concentrations of EA in the sclerotia can vary greatly and consequently affect EA consumption by individual animals. Blaney et al. (2011) showed that steers fed 1.1 mg of sorghum EA/kg of diet DM had a 19% reduction in intake and 26% reduction in ADG compared to the control steers, and that this decline was even greater when the ambient temperatures rose above 21.1 °C. Coufal-Majewski et al. (2017b), continuously fed EA diets ranging from 0.93g to 2.45 mg/kg EA to rams, and reported that there was a linear decrease in ADG with increasing EA. Similar to the present study, Coufal-Majewski et al. (2017b) also reported an increased rectal temperature of 0.33 °C in EA-fed rams compared to controls. In the current study, EA consumption resulted in significant reductions in DMI, ADG, and shrunk total BW gain, and increased rectal temperature. These effects of EA on beef steers suggest that the feeding 2 mg/kg EA does have a negative impact that will not only reduce growth performance but also increase body temperature.

The consumption of 2 mg of EA/kg of diet DM consistently reduced DMI throughout the entire study, leading to reduced ADG, lower final BW and quality grades. Koontz et al. (2015) also concluded that cattle consuming ergot alkaloids composed mainly of ergovaline or ergotamine gained less as a result of reduced feed intake. The data from the present study also agrees with Sarich et al. (2023) where beef steers fed concentrations of cereal EA up to 3.00 mg/kg had reduced ADG, shrunk final BW, and shrunk total BW gain. However, these findings contrast with Stanford et al. (2022) where no significant negative effects were observed on shrunk weight, DMI, ADG, or rectal temperature of BG steer fed 1.75 mg of EA/kg of diet DM in a pellet or mash. Likewise, Grusie et al. (2018) did not observe a reduction in weight gain or growth performance of beef cows fed 0.822 mg EA/kg of diet DM. Although both authors reported no effect on intake and growth performance, Stanford et al. (2022) did report some minor effects on select blood serum parameters. This contrasting results are likely due to the lower concentrations of total EA fed by Grusie et al. (2018) and Stanford et al. (2022) compared to the present study and possibly due to differences in the profile of alkaloids fed. Rectal temperature increases due to EA consumption have been reported multiple times (Coufal-Majewski et al., 2017b; Cowan et al., 2019; Sarich et al., 2023). The increase in rectal temperature due to EA consumption is believed to be associated with alkaloid induced peripheral vasoconstriction affecting the animal’s ability to thermoregulate (Stanford et al., 2018). Gaughan et al. (2010) showed that an increase in heat load and body temperature decreased DMI and growth performance of unshaded steers compared to shaded steers. The increased rectal temperature promoted by EA may also be one of the contributing factors to the reduction in DMI observed for EA fed steers.

As this study was one of the first to intermittently feed EA to beef cattle, the findings provide insight that the effects of EA may last longer than 2 wk, and the length of time required for the DMI and ADG of steers to recover from EA exposure should be further evaluated. Multimyography of the lateral saphenous vein and Doppler ultrasonography of the caudal artery studies in cattle fed EA from endophyte-infected tall fescue (ergovaline + ergovalinine) showed that the vasoconstriction promoted by EA was not completely recovered even after 4 wk of cattle removal from infected pastures (Klotz et al., 2016). They suggested that a minimum of 5 to 6 wk was needed for complete vascular recovery after EA exposure. This suggests that the prolonged effects of EA induced vasoconstriction may last multiple weeks longer than the 2 wk EA-free intermittent period provided in this current study, and that a longer period without EA in the diet in future intermittent feeding studies is warranted to better understand its impact on intake and growth performance.

The use of heat and pressure during pelleting has been suggested to alter EA chemical bonds and potentially reduce the toxicity of EA (Coufal-Majewski et al., 2016; Stanford et al., 2022). The growth performance of both BG and FN steers did not differ between the ergot mash or pelleted treatment for DMI or ADG, with the exception of gain:feed of steers in the intermittent EA mash diet during the FN phase which increased compared to all other treatments. Opposite results were observed in lambs fed pelleted EA diets compared to EA mash diets (up to 0.43 mg of total EA/kg of diet DM), with an increase in gain:feed and ADG of lambs fed pelleted diets (Coufal-Majewski et al., 2017a). Different than the present study, the pelleting process did not increase the measured total EA concentration compared to their mash diet (Coufal-Majewski et al., 2017a). Differences between sheep and cattle in sorting behavior and in the diets fed and the lower levels of total EA fed by Coufal-Majewski et al. (2017a) may be responsible for these contrasting results. Similar to Stanford et al. (2022) using beef steers, the present study did not find a decrease in EA or toxicity as result of pelleting.

### Ear, tail, and hoof temperature

Infrared thermography can provide an indirect assessment of body temperature and blood flow to the extremities (Farrar et al., 2020). A similar protocol described by Stanford et al. (2022) for infrared thermography of the ears, coronary band, and tail-head was used in the present study in both BG and FN phases. Stanford et al. (2022) reported no significant effect of cereal EA on surface temperatures of the ear, coronary band, or tail when included in the diet at 1.75 mg/kg of DM for BG beef steers. In the current study, some evidence of peripheric vasoconstriction promoted by EA was observed in the BG phase by a tendency to reduce ears temperature (period 4) and in the FN phase by a decrease in tail temperature (periods 1 and 2) and a tendency to decrease hoof temperature (periods 4 and 6). Evidence of temperature changes in the ears of beef steers was also reported by Sarich et al. (2023), where EA promoted a linear decrease in ear temperature during BG and a linear increase during FN phase of steers receiving up to 3.0 mg EA/kg diet. Greater inclusion of EA in the diet of feedlot steers in the study conducted by Sarich et al. (2023) likely promoted more pronounced changes in thermoregulation, as in the present study we only observed a minor physiological effect of EA (2.0 mg/kg). Although not consistent, differences were also observed in the present study for steers fed EA in a mash compared to a pellet form and they may be related to sorting behavior of the steers, although not measured in the present study. However, this inconsistency may also be attributed to the accuracy of thermographic images that can be influenced by hair coat of the cattle, cleanliness, and image inconsistency and distance during capturing, with increased hair coat and reduced cleanliness likely during extreme cold or warm temperatures. Direct measurement of vasoconstriction using Doppler ultrasonography of the caudal artery as described by Klotz et al. (2016) may be a more sensitive method.

The fact that the present study was conducted between early October and late June, may account for the lack of EA induced heat stress in steers. Cattle in the present study were also sheltered from the sun instead of being in open feedlot pens as in the study of Sarich et al. (2023). However, temperatures below freezing can also increase risk of cold stress, gangrenous symptoms, heat loss, and vasoconstriction (Cowan et al., 2018; Wang et al., 2023). Although the study was conducted during temperatures as low as −35.3 °C between the winter months of November to February, the effects of EA on the extremities were minimal which could be a result of the concentrations of EA in the diets tested not being high enough (2.0 mg total EA/kg of DM) as compared to Sarich et al. (2023) who fed steers diets with EA up to 3 mg/kg of DM.

### Hematology, blood and hair chemistry

Complete blood count is a valuable analysis to assess health and disease of cattle and other mammals (Alfaro et al., 2021). The results of the current study fit within the ranges of the normal hematology of beef cattle as reported by Jones and Allison (2007). There were few treatment effects observed for CBC. During BG, a decrease in RBC for steers fed EA continuously vs. intermittently was observed, with no effects on WBC, granulocytes, lymphocytes, monocytes, or platelets. Likewise, no treatment effects were observed for CBC during the FN period. Interestingly, Stanford et al. (2022) reported low WBC counts including low monocytes and granulocytes for BG steers fed EA at 1.75 mg/kg. They also reported that hemoglobin concentrations were reduced in EA fed steers, but the hematocrit was not affected as the RBC count was increased (Stanford et al., 2022). Different sources of ergot and EA profiles, and levels fed by Stanford et al. (2022) were likely responsible for differences between studies.

Since mycotoxins are metabolized in the liver (Coufal-Majewski et al., 2016), multiple blood chemistry parameters and liver enzymes may be affected by EA. Intermittently feeding EA compared to continually resulted in lower blood serum values for ALB, GLOB, Amylase, GGT and Ca during the BG phase, perhaps due to a potential liver metabolism adaptation of continuously fed EA steers. These differences were not observed during the FN phase. Reduced ALB and GLOB concentrations in the blood of the EA fed steer is indicative of impaired liver function and has also been associated with the presence of liver abscess (Macdonald et al., 2017). Interestingly, ALP levels decreased in the BG phase of the current study when steers where fed EA similarly to what has been reported by Jackson et al. (2015) and Sarich et al. (2023), which may be associated with decreased DMI in EA fed steers. In the FN phase, a reduction in AMYL and BUN was observed and they both have been associated with protein status in cattle (Smock et al., 2023), which may be the result of decreased DMI and BW gain in EA feed steers. Lastly, as lipase is produced in the adipose and muscle tissue (Ren et al., 2002), the decrease in values observed in the FN phase may be associated with reduced BW gain and fatness in EA fed steers.

Hair cortisol concentrations in the present study were not affected by EA compared to controls during BG, similar to Stanford et al. (2022). Contrary to expectations, during the FN hair cortisol concentrations decreased for all EA diets in the present study. In contrast, Sarich et al. (2023) showed a tendency for a quadratic response with increasing concentrations of cereal EA in the diet of backgrounding feedlot steers, where the control steers (no EA in the diet) and the highest level of EA fed (3 mg/kg) displayed the lowest hair cortisol values. They did not observe differences in hair cortisol in the FN phase. Increase in hair cortisol has been used as a marker for long-term stress in cattle (Moya et al., 2013). The reason for the decrease in hair cortisol of EA fed steers in the FN phase of the current study is unclear as the relationship between hair cortisol and exposure to ergot is not well described. It is possible that the lower DMI observed for steers fed EA diets increased ruminal pH and reduced the occurrence of subacute ruminal acidosis in the finishing phase, consequently decreasing hair cortisol levels (Moya et al., 2015). Additionally, EA may also bind to adrenergic receptors and act as antagonist, consequently reducing the levels of cortisol production.

Exposure of cattle to EA can reduce concentrations of plasma prolactin by activating dopamine receptors that reduce prolactin production in the anterior pituitary (Paterson et al., 1995; Schardl et al., 2006; Klotz, 2015). These alkaloids may also act on the hypothalamus, which regulates the release of prolactin inhibiting hormone via dopamine, thus preventing the release of prolactin (Burfening, 1994; Poole and Poole, 2019). In the current study, feeding diets with EA did not reduce serum prolactin levels in either BG or FN steers. Similar results have been shown by Grusie et al. (2018), Cowan et al. (2018), and Sarich et al. (2023) with cereal ergot alkaloids. However, these contrast with other cereal EA studies such as Cowan et al. (2023) who observed a decrease in prolactin concentration in breeding bulls fed EA at 1.13 and 2.23 mg/kg. A reduction in prolactin has also been observed in sheep, where cereal grain EA resulted in a linear decrease in blood serum prolactin as EA concentrations in the diet increased (Coufal-Majewski et al., 2017a, 2017b). The lack of change in serum prolactin in the present study could be attributed to many environmental and physical factors such as stress, temperature, photoperiod, and animal handling (Cowan et al., 2018; Sarich et al., 2023). As the study began in October and ran until the beginning of June, and the day length increased from the end of December to June, this could have promoted prolactin production countering the negative impact of EA on prolactin production. This increase in day length has been shown to increase serum prolactin concentration in Holstein heifers (Peters and Tucker, 1978). Not only does the photoperiod change the circulating concentrations of prolactin but it also affects the expression of the prolactin receptor (Auchtung and Dahl, 2004). However, this outcome in our study may also be a result of the lack of sensitivity of the bovine prolactin ELISA kit used. Both Stanford et al. (2022) and Sarich et al. (2023) used the same ELISA kit as we did and reported no effect of EA, which is surprising given the marked reductions in performance of cattle fed EA in the current study and that of Sarich et al. (2023). The radioimmunoassay (RIA) technique has been considered a more sensitive and reliable method for prolactin analysis (Blaney et al., 2011; Cowan et al., 2023).

### Carcass characteristics

Mixed results have been reported about the impact of EA consumption on carcass characteristics of sheep and beef cattle (Schumann et al., 2007; Coufal-Majewski et al., 2017a; Sarich et al., 2023). In accordance with the current study, recent data from Sarich et al. (2023) showed a reduction in carcass weight, dressing percentage, and Canadian AA quality grade, and increase in Canadian A quality grade with increasing concentrations of EA in the diet. However, in contrast to the present study, Schumann et al. (2007) did not observe any impact on growth performance or carcass traits when feeding 0.42 mg of EA/kg of DM to growing Holstein bulls. Similarly, in a study using lambs, Coufal-Majewski et al. (2017a) did not observe an effect of feeding EA on hot carcass weight, dressing percentage or other carcass traits with EA at a maximum of 0.35 mg/kg of diet. However, in the present study, the ribeye area, lean meat yield, yield score, and Canadian AAA grade of carcasses were all negatively affected by EA. Conversely, Coufal-Majewski et al. (2017b) showed no impact on hot carcass weight or grade with rams continuously fed EA ranging from 0.93 to 2.4 mg/kg of diet DM with no carcass condemnations found at the abattoir. Perhaps cattle are more sensitive to EA than feeder lambs, as for all cattle studies feeding 2 mg/kg or greater concentrations of EA resulted in significant carcass impacts.

## Conclusion

Pelleting increased concentrations of measured EA in the diet supplement compared to a mash form although toxicity did not change. Analysis of EA in complete diets returned unrealistically low levels that did not align with the total EA in the ergot screenings used to make the diet supplements and inclusion of the supplements in the diets. Feeding feedlot steers 2 mg of cereal EA/kg of diet DM resulted in an approximately 10% reduction in both intake and weight gain, a response that was consistent during the BG and FN phases, leading to a reduction in carcass weight and fatness. Steers fed EA diets had increased rectal temperatures (+0.2 to 0.4 °C) suggesting that thermoregulation was compromised, although no classical symptoms of heat stress were observed. Environmental temperatures were not high enough to induce heat stress in steers, but temperatures did drop below −30 °C for a few days, which may have increased the impact of EA on peripheral vasoconstriction. The inclusion of EA in the diet either continuously or intermittently reduced feed intake and growth performance of beef steers resulting in carcass weight losses ranging from 6 to 25 kg, and in the reduction of carcasses classified as AAA quality grade by 30% to 50%. Inclusion of EA in the diets also promoted a slight reduction in blood serum albumin, globulin, and alkaline phosphatase during the BG phase, and in amylase, lipase, and urea nitrogen in the FN phase, but these values were still in the normal range described for cattle. Removing EA from the diet for 2 wk of a 3-wk period from the beginning of the backgrounding period to the end of finishing period did not allow steers to exhibit sufficient compensatory gain to recover from 1 wk of exposure to dietary EA, although the recovery period may depend on the concentrations of EA fed. Pelleting did not reduce the impact of EA on any of the measured parameters nor was it an effective method of reducing EA toxicity.

## Abbreviations

ADF: acid detergent fibre
ADG: average daily gain
ALB: albumin
ALP: triglycerides
ALKP: alkaline phosphatase
AMYL: amylase
ALT: alanine aminotransferase
BUN: blood urea nitrogen
BG: backgrounding
BW: body weight
Ca: calcium
CREA: creatinine
CFIA: Canadian Feed Inspection Agency
CON: control ergot mash
CEM: continuous ergot mash
CP: crude protein
DM: dry matter
DMI: dry matter intake
DOF: days on feed
EA: ergot alkaloids
FN: finishing
GGT: gamma-glutamyl transpeptidase
G: F, gain efficiency
GLOB: globulin
GLU: glucose
HCW: hot carcass weight
IEM: intermittent ergot mash
IEP: intermittent ergot pellet
LMY: lean meat yield
LIPA: lipase
NDF: neutral detergent fiber
RBC: red blood cells
SD: standard deviation
TAG: triglycerides
TBIL: total bilirubin
TP: total protein
TMR: total mixed ration
WBC: white blood cells
